# Multi-atlas label fusion with random local binary pattern features: Application to hippocampus segmentation

**DOI:** 10.1038/s41598-019-53387-9

**Published:** 2019-11-14

**Authors:** Hancan Zhu, Zhenyu Tang, Hewei Cheng, Yihong Wu, Yong Fan

**Affiliations:** 1 School of Mathematics Physics and Information, Shaoxing University, Shaoxing, Zhejiang 312000 China; 2 Beijing Advanced Innovation Center for Big Data and Brain Computing, Beihang University, Beijing, 100191 China; 3 Department of Biomedical Engineering, School of Bioinformatics, Chongqing University of Posts and Telecommunications, Chongqing, 400065 China; 4 National Laboratory of Pattern Recognition, Institute of Automation, Chinese Academy of Sciences, Beijing, 100190 China; 5 Department of Radiology, Perelman School of Medicine, University of Pennsylvania, Philadelphia, PA 19104 USA

**Keywords:** Image processing, Machine learning

## Abstract

Automatic and reliable segmentation of the hippocampus from magnetic resonance (MR) brain images is extremely important in a variety of neuroimage studies. To improve the hippocampus segmentation performance, a local binary pattern based feature extraction method is developed for machine learning based multi-atlas hippocampus segmentation. Under the framework of multi-atlas image segmentation (MAIS), a set of selected atlases are registered to images to be segmented using a non-linear image registration algorithm. The registered atlases are then used as training data to build linear regression models for segmenting the images based on the image features, referred to as random local binary pattern (RLBP), extracted using a novel image feature extraction method. The RLBP based MAIS algorithm has been validated for segmenting hippocampus based on a data set of 135 T1 MR images which are from the Alzheimer’s Disease Neuroimaging Initiative database (adni.loni.usc.edu). By using manual segmentation labels produced by experienced tracers as the standard of truth, six segmentation evaluation metrics were used to evaluate the image segmentation results by comparing automatic segmentation results with the manual segmentation labels. We further computed Cohen’s d effect size to investigate the sensitivity of each segmenting method in detecting volumetric differences of the hippocampus between different groups of subjects. The evaluation results showed that our method was competitive to state-of-the-art label fusion methods in terms of accuracy. Hippocampal volumetric analysis showed that the proposed RLBP method performed well in detecting the volumetric differences of the hippocampus between groups of Alzheimer’s disease patients, mild cognitive impairment subjects, and normal controls. These results have demonstrated that the RLBP based multi-atlas image segmentation method could facilitate efficient and accurate extraction of the hippocampus and may help predict Alzheimer’s disease. The codes of the proposed method is available (https://www.nitrc.org/frs/?group_id=1242).

## Introduction

Accurate and automatic hippocampus segmentation from magnetic resonance (MR) brain images is important in several neuroimaging studies of brain disorders, such as brain cancer, epilepsy, and Alzheimer’s disease (AD)^1–3^. To achieve fully automated hippocampus segmentation, atlas-based methods have been proposed for hippocampus segmentation^4^. These methods typically adopt an atlas image with a manually labeled hippocampus and use a nonlinear image registration algorithm to align the atlas to the image to be segmented, referred to as a target image hereafter, and the segmentation of the target image is then achieved by propagating the atlas label to the target image space. However, there may be a limit to the performance of these techniques for a case in which there exists a large anatomic difference between the atlas and target images, which would make the image registration difficult.

To partially circumvent this problem, multi-atlas image segmentation (MAIS) methods have been proposed^5,6^. In contrast to the single-atlas image segmentation method, the MAIS methods generally comprise image registration and label fusion. In MAIS methods, each atlas image is first registered to the target image and the atlas label is propagated to the target image space accordingly^7^. The propagated label maps are then fused to get a final segmentation^8^. In some MAIS methods, a small number of atlas images that are better matched with the target image are selected instead of using all the available atlas images^9–12^.

In the MAIS methods, the label fusion step is a core component as it fuses the propagated atlas labels to obtain the image segmentation result. Several label fusion strategies have been developed, such as majority voting^6^ and weighted voting^13^. For better accounting for the image registration errors, nonlocal patch-based (NLP) methods were proposed^14,15^. In the NLP methods, for labeling a target image voxel a number of voxels in the searching region in each atlas image are considered and high weight factors are assigned to those more similar to the target image voxel. To improve the accuracy and robustness of the NLP methods, several label fusion methods have been proposed, including sparse representation^16,17^, dictionary learning^18^, manifold learning^19–22^, and joint label fusion (JLF)^23^. In addition to these methods, NLP methods have also been combined with statistical label fusion methods^5^, which have been highly successful^24–26^.

Recently, pattern recognition based label fusion methods have been developed and successfully applied to a variety of medical image segmentation problems^10,27–30^. These methods solve the image segmentation problem as a pattern recognition problem by considering registered atlas images as training data in order to build pattern recognition models for predicting the segmentation labels of images to be segmented. It has been demonstrated that feature extraction is important in pattern recognition based label fusion methods^10,27^. For example, image intensity and texture image features were adopted to train support vector machine (SVM) classifiers for predicting segmentation labels^10,27^, the random forest classification algorithm was adopted to construct classifiers for label fusion using local and contextual image features^28,29^, artificial neural networks were built for label fusion using statistical and textural features^30^. The studies on the aforementioned methods have demonstrated that MAIS algorithms could achieve improved performance by building pattern recognition models using rich image features.

In this paper, we propose a novel feature extraction method based on local binary pattern (LBP) features^31,32^, referred to as random local binary pattern (RLBP), for building linear regression models to achieve reliable and accurate label fusion in MAIS. We have illustrated that the proposed RLBP method is more robust to image noise than the LBP method and is capable of capturing discriminative information for the image segmentation. Our method is validated for segmenting hippocampi from MR brain images. In the validation experiment, we compared the proposed RLBP method with the LBP method^32^. The results showed that our method could provide more accurate segmentation results than the LBP method. We also compared our method with state-of-the-art label fusion methods, including NLP^14,15^, local label learning (LLL)^10^, JLF^23^, and nonlocal weighted voting with metric learning (NLW-ML)^33^. The results demonstrated that our RLBP method was competitive to state-of-the-art label fusion methods. In addition, we also performed a hippocampal volumetric analysis experiment. The obtained results demonstrated that our RLBP method performed well in detecting the volumetric differences of the hippocampus between AD, mild cognitive impairment (MCI), and normal control (NC) groups. Part of this work has been previously presented in^34^.

## Materials and Methods

### Imaging data

A dataset comprising 135 T1 MRI scans with manually labeled hippocampi from the Alzheimer’s Disease Neuroimaging Initiative (ADNI) database (adni.loni.usc.edu/) was used for validating the proposed algorithm. The ADNI was launched in 2003 as a public-private partnership, led by Principal Investigator Michael W. Weiner, MD. The primary goal of ADNI has been to test whether serial magnetic resonance imaging, positron emission tomography, other biological markers, and clinical and neuropsychological assessment can be combined to measure the progression of mild cognitive impairment and early Alzheimer’s disease. For up-to-date information, see www.adni-info.org. The ADNI MRI scans were acquired using a sagittal 3D MP-RAGE T1-w sequence (TR = 2400 ms, minimum full TE, TI = 1000 ms, FOV = 240 mm, voxel size of $1.25\times 1.25\times 1.2{{\rm{mm}}}^{3}$).

### Manual labeling of hippocampus

**Figure 1:**
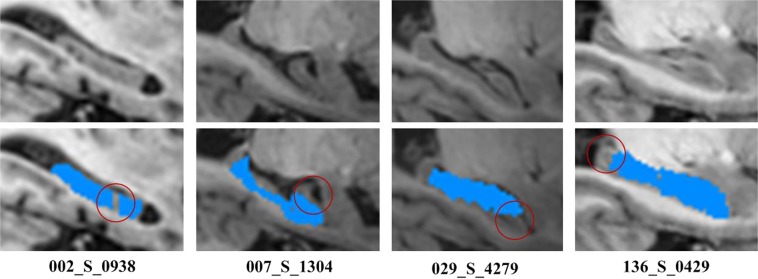
MR brain images (top row) with problematic hippocampus labels (bottom row).

Hippocampus labels of the image data in the Neuroimaging Informatics Technology Initiative format were provided by the European Alzheimer’s Disease Consortium and Alzheimer’s Disease Neuroimaging Initiative harmonized segmentation protocol (EADC–ADNI)^35^, which can be publicly downloaded (www.hippocampal-protocol.net). The dataset consists of a preliminary release part with 100 subjects and a final release part with 35 subjects. In the preliminary release part, one subject’s hippocampus label missed several slices. In the final release part, the hippocampus labels and the images of three subjects are not well matched. Their subject identification numbers are 002_S_0938, 007_S_1304, 029_S_4279 and 136_S_0429 respectively. Such problems might be caused by imaging data format conversion. Figure 1 shows these MR brain scans and their corresponding hippocampus labels. We used the remaining 32 subjects in the final release part as a training data set and the remaining 99 preliminary release subjects as a testing data set in the present study.

The training data set contains 14 NC, 11 MCI, and 7 AD subjects (see Table 1). The testing data set contains 29 NC, 34 MCI, and 36 AD subjects (see Table 2). The testing MCI subjects were further classified as stable MCIs (sMCI, n = 11) and progressive MCIs (pMCI, n = 21), according to the ADNI clinical data downloaded on July 17, 2017. However, 2 MCI subjects could not be assigned to either sMCI or pMCI groups due to missing data.

**Table 1:** Demographic and diagnostic information of the training subjects.

	NC	MCI	AD
Number of subjects	14	11	7
Age (years): mean ± std	76.44 ± 9.014	77.01 ± 9.23	77.09 ± 8.31
Males/Females	5/9	6/5	1/6

**Table 2:** Demographic and diagnostic information of the testing subjects.

	NC	MCI	AD
Number of subjects	29	34	36
Age (years): mean ± std	75.79 ± 6.72	74.24 ± 7.67	73.70 ± 8.18
Males/Females	16/13	20/14	20/16

All the MR brain images were aligned along the line that passes through the anterior and posterior commissures of the brain, corrected for their bias field, and finally spatially normalized to the MNI152 template space using affine transformation^35^.

### Atlas selection and registration

To reduce the computation cost and improve the registration accuracy, we identified two bounding boxes, one for the left hippocampus and the other for the right hippocampus. In particular, the sizes of the obtained bounding boxes were $48\times 77\times 67$ and $47\times 70\times 66$ for the left and right hippocampi respectively, which were large enough for covering the hippocampi of an unseen target image^10,36^. To improve the segmentation performance, we selected $N$ atlases which were most similar to the target image based on normalized mutual information of the image intensities within the bounding box^9^. Then, we used a nonlinear, cross-correlation-driven image registration algorithm to register these atlas images to the target images to achieve a better local anatomy matching between the target image and each atlas image^37^. To further reduce the computation cost, the majority voting label fusion was performed to get an initial segmentation for the target image. We then applied the proposed method introduced in the following two subsections to segment voxels which did not receive a unanimous vote in the majority voting label fusion^10^.

### Machine learning based MAIS

With atlas selection and image registration, we have $N$ warped atlases ${A}_{i}=({I}_{i},{L}_{i}),\,i=1,2,\ldots ,N$, where ${I}_{i}$ is the *i*-th warped atlas image and ${L}_{i}$ is its warped label map. A machine learning based MAIS method is to infer the label map of the target image $I$ by building prediction models based on the warped atlases such that the image segmentation problem is solved as a pattern recognition problem.

To label the target voxel *x*, a set of training voxels is identified in a cube-shaped searching neighborhood $N(x)$ of size $(2{r}_{s}+1)\times (2{r}_{s}+1)\times (2{r}_{s}+1)$ of the corresponding voxel $X$ from all atlas images, and a feature vector for each of the training voxels is extracted to characterize the local image information. By denoting the feature vector of the target voxel by ${f}_{x}$, the feature vector of voxel $j$ of the ${i}^{th}$ atlas by ${f}_{i,j}$ and its corresponding segmentation label by ${l}_{i,j}\in \{-1,1\}$ where 1 represents the region of interest and −1 represents the background, we obtain a training set ${D}_{x}=\{({f}_{i,j},{l}_{i,j})|i=1,\mathrm{..},N,\,j\in N(x)\}$. A pattern recognition model is finally built by using the training set ${D}_{x}$ to predict the segmentation label of the test sample ${f}_{x}$.

### RLBP feature extraction method

**Figure 2:**
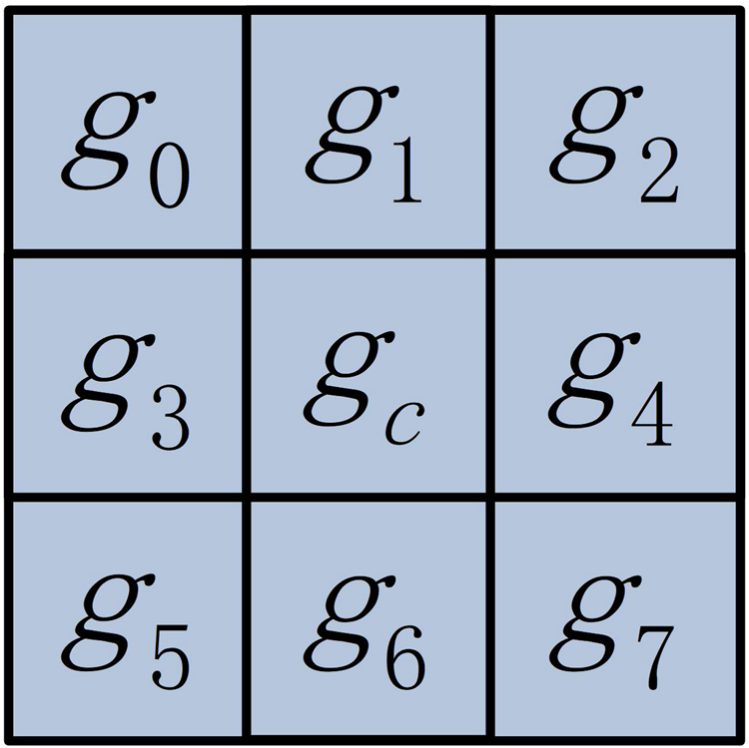
Illustration of the image neighborhood for computing LBP features.

To characterize each image voxel, a new feature extraction method is developed based on the LBP image feature extraction method^32^. In particular, the LBP image feature extraction method was developed for 2D images. Given a $3\times 3$ neighboring system as illustrated by Fig. 2, the LBP features are computed as

1 $$LB{P}_{p}=s({g}_{p}-{g}_{c}),\,p=0,\ldots ,7,$$

where $$s(x)=\{\begin{array}{c}1,\,x\ge 0\\ 0,\,x < 0\,\end{array}$$, and ${g}_{c}$ and ${g}_{p}$ are the image intensity values of the center pixel and its neighboring voxels respectively. The binarization of the local image intensity difference makes LBP features robust to illumination and image contrast variations. However, it is sensitive to image noise^38^.

**Figure 3:**
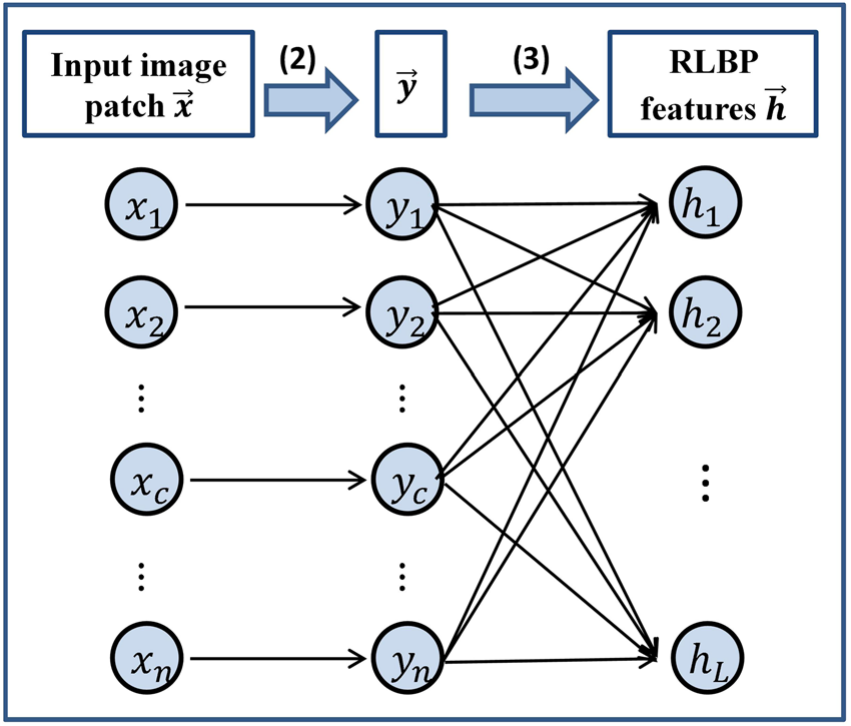
Illustration of the computation of RLBP features.

In order to obtain more discriminative and robust image features, we propose an RLBP feature extraction method, as illustrated in Fig. 3. First, we extend the LBP method to be applicable to 3D images. Thus, for a voxel $C$ in a 3D image with a cubic image patch centered at itself with $(2{r}_{p}+1)\times (2{r}_{p}+1)\times (2{r}_{p}+1)$ voxels, a difference image intensity vector is computed using

2 $$\overrightarrow{y}={[{x}_{1}-{x}_{c},{x}_{2}-{x}_{c},\ldots ,{x}_{n}-{x}_{c}]}^{T},\,n={(2{r}_{p}+1)}^{3},$$

where ${x}_{i},\,i=1,\ldots ,n$ is the image intensity value of a voxel in the cubic image patch, and ${x}_{c}$ is the image intensity value of the voxel $c$.

Then, we constructed a large number of random transformation functions to generate RLBP features with the following formula,

3 $$h(\cdot )={[{h}_{1}(\cdot ),{h}_{2}(\cdot ),\ldots ,{h}_{L}(\cdot )]}^{T}\in {R}^{L},$$

where $${h}_{j}(\overrightarrow{y})=s({\overrightarrow{w}}_{j}\cdot \overrightarrow{y})=\{\begin{array}{c}1,\,{\overrightarrow{w}}_{j}\cdot \overrightarrow{y}\ge 0\\ 0,\,{\overrightarrow{w}}_{j}\cdot \overrightarrow{y} < 0\end{array},\,j=1,\ldots ,L$$, “·” represents the dot multiplication of vectors, and ${\overrightarrow{w}}_{j}\in {R}^{n}$ is a random vector whose values are uniformly distributed in $[-1,1]$. Using the RLBP feature extraction method, a feature vector $\overrightarrow{f}(c)=h(\overrightarrow{y})$ is obtained for the given voxel $c$.

**Figure 4:**
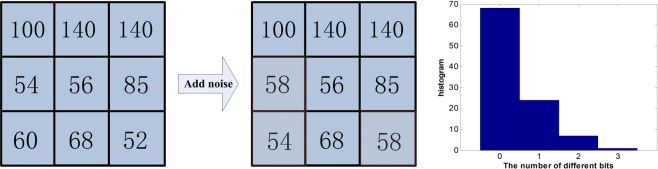
Example image patch, the version of it that is corrupted by noise, and the histogram of the differences between the RLBP features, for 100 computations.

The LBP method obtains the binarized values directly from the sign of the differences between the adjacent pixels and the center pixel regardless of the absolute value of the differences. It has been demonstrated that small pixel difference is vulnerable to noise^38^. In contrast to the LBP method, the proposed RLBP method adopts a large number of random weights to obtain weighed sums of the image difference vectors before the binarization. The random combination processing increases the robustness of the image difference as illustrated by the example shown in Fig. 4. Particularly, Fig. 4 shows a 2D image patch and a version of it that is corrupted by noise. Their LBP features are ($$01110111$$) and ($$11011111)$$, which are different in 3 out of 8 bits. By setting $${\rm{L}}=20$$ (the dimension of the generated RLBP feature is 20) for 100 computations, the RLBP features of these two image patches are the same in 68 computations, different in 1 bit in 24 computations, different in 2 bits in 7 computations, and different in 3 bits in 1 computation, thus illustrating that the RLBP features are statistically more robust to image noise than the LBP features.

### Linear regression with RLBP features for label fusion

Based on the generated RBLP features ${\overrightarrow{f}}_{i,j}$ and the corresponding label ${l}_{i,j}$, we use the following linear regression model to predict the label of target voxel,

4 $${{\rm{argmin}}}_{\overrightarrow{\beta }}F(\overrightarrow{\beta })=\frac{1}{2}{\Vert \overrightarrow{\beta }\Vert }^{2}+\frac{1}{2}C\sum _{i,j}{({l}_{i,j}-{\overrightarrow{\beta }}^{T}{\overrightarrow{f}}_{i,j})}^{2},$$

where $\Vert \cdot \Vert$ is the L2 norm and $C$ is a balance parameter between the data fitting cost and the regularization term.

To solve the optimization problem of Eq. (4), we let the gradient of $F(\overrightarrow{\beta })$ be equal to zero,

5 $$\nabla F(\overrightarrow{\beta })=\overrightarrow{\beta }+{\rm{C}}\sum _{i,j}({l}_{i,j}-{\overrightarrow{\beta }}^{T}{\overrightarrow{f}}_{i,j})\cdot (-{\overrightarrow{f}}_{i,j})=\overrightarrow{0}.$$

By reorganizing Eq. (5), we obtain

$$(\frac{I}{C}+\sum _{i,j}\,{\overrightarrow{f}}_{i,j}{\overrightarrow{f}}_{i,j}^{T})\overrightarrow{\beta }=\sum _{i,j}\,{l}_{i,j}\,{\overrightarrow{f}}_{i,j},$$

where $I$ is an identity matrix. We then obtain

$$\overrightarrow{\beta }={(\frac{I}{C}+\sum _{i,j}{\overrightarrow{f}}_{i,j}{\overrightarrow{f}}_{i,j}^{T})}^{-1}(\sum _{i,j}{l}_{i,j}\,{\overrightarrow{f}}_{i,j}),$$

and estimate the label of the target voxel as

6 $$\overrightarrow{L}(x)={\rm{sgn}}({\overrightarrow{\beta }}^{T}{\overrightarrow{f}}_{x}).$$

### Parameter optimization

There are five parameters in our method, including the number of selected atlases ($N$), the dimension of the generated RLBP feature $(L)$, balance parameter $(C)$ in the linear regression model, search radius $({r}_{s})$, and patch radius $({r}_{p})$. We first chose the best value of $N$ from {5, 10, 15, 20, 25, 30} using the baseline majority voting label fusion method. We fixed the searching radius to ${r}_{s}=1$, since a nonlinear image registration algorithm was used to warp the atlas images to the target image ^10,33^, and selected the best value of $L$ from {100, 500, 1000, 1500, 2000}. Finally, we determined the other two parameters $C$ and ${r}_{p}$ empirically from {${4}^{-5},{4}^{-4},\ldots ,{4}^{0}$} and $\{1,2,3,4,5\}$ using a grid-searching strategy. We performed leave-one-out cross-validation experiments based on the training set for optimizing these parameters.

### Evaluation metrics for segmentation results

The segmentation accuracy of the proposed method was evaluated by using the test dataset. We evaluated the image segmentation results using six segmentation evaluation metrics that measure differences between the automatic segmentation results and their corresponding manual segmentation labels in different aspects, including Dice coefficient (Dice), Jaccard, Precision, Recall, volume difference (dVol), and mean distance (MD). These metrics are defined as,

$${\rm{Dice}}=2\frac{{\rm{V}}({\rm{A}}\cap {\rm{B}})}{{\rm{V}}({\rm{A}})+{\rm{V}}({\rm{B}})},\,{\rm{Jaccard}}=\frac{{\rm{V}}({\rm{A}}\cap {\rm{B}})}{{\rm{V}}({\rm{A}}\cup {\rm{B}})},$$

$${\rm{Precision}}=\frac{{\rm{V}}({\rm{A}}\cap {\rm{B}})}{{\rm{V}}({\rm{B}})},\,{\rm{Recall}}=\frac{{\rm{V}}({\rm{A}}\cap {\rm{B}})}{{\rm{V}}({\rm{A}})},$$

$${\rm{dVol}}=|V(A)-V(B)|,\,{\rm{MD}}={{\rm{mean}}}_{e\in \partial A}(mi{n}_{f\in \partial B}d(e,f)),$$

where $A$ is the manual segmentation, $B$ is the automated segmentation, $\bar{A}$ and $\bar{B}$ are the complements of $A$ and $B$, $V(X)$ is the volume of $X$, $\partial A$ is a set of the boundary voxels of $A$, and $d(\cdot ,\cdot )$ is the Euclidian distance between two voxels.

The correlation coefficients between the hippocampal volumes estimated using the manual segmentation and the automatic segmentation methods were also computed.

### Comparison of the proposed method with state-of-the-art algorithms

The proposed method was compared with state-of-the-art label fusion methods, including NLP^14^, LLL^10^, JLF^23^ and NLW-ML^33^. The parameters of all these methods were also optimally selected according to the results of leave-one-out cross-validation experiments on the training set. As in the case of the RLBP, the searching radius *r*_*s*_ was fixed as 1 (searching neighborhood *V* with a size of 3 × 3 × 3) for all these methods.

The NLP method comprises two additional parameters, which include patch radius ${r}_{p}$ and ${\sigma }_{x}$ in the Gauss function. ${\sigma }_{x}$ was set as ${\sigma }_{x}=mi{n}_{{x}_{s,j}}\{{\Vert P(x)-P({x}_{s,j})\Vert }_{2}+\varepsilon \},\,s=\mathrm{1..}N,\,j\in V$, where $\varepsilon =1e-20$ used to ensure numerical stability^14,15^. The best value of ${r}_{p}$ was selected from {1, 2, 3, 4}. For the LLL method, the parameter C in the sparse linear SVM classifiers was set to its default vaule (C = 1). The patch radius ${r}_{p}$ and the number of training samples $k$ were determined from {1, 2, 3, 4} and {300, 400, 500} using the grid-searching strategy. For the JLF method, the patch radius ${r}_{p}$ and parameter $\beta$ were determined from {1, 2, 3} and {0.5, 1, 1.5, 2} using the grid-searching strategy. For the NLW-ML method, the best values of two parameters ${r}_{p}$ and $k$ were selected from {1, 2, 3} and {3, 9, 27} using the grid-searching strategy.

The proposed method (RLBP) was also compared with LBP features for label fusion using the same linear regression model to illustrate the effectiveness of the proposed RLBP feature extraction method. As the only difference between the RLBP and LBP methods is that a large number of random combinations are used in the RLBP method before binarization, parameters of the same values were used in the LBP method as RLBP.

### Hippocampal volumetric analysis

A hippocampal volumetric analysis was performed based on the test dataset. As the hippocampal volume varies with the brain size, we corrected the hippocampal volumes according to the intracranial volumes estimated using SPM12 (http://www.fil.ion.ucl.ac.uk/spm/). The hippocampal volumes were then corrected using the following equation

7 $${\rm{Corrected}}\,{\rm{volume}}={\rm{Measured}}\,{\rm{volume}}\times \frac{{{\rm{ICV}}}_{mean}}{ICV},$$

where ICV is the intracranial volume of the testing subject whose hippocampal volume is to be corrected, and ${{\rm{ICV}}}_{mean}$ is the mean intracranial volume of all testing subjects. All volumes in formula (7) were measured in cm^3^.

In order to investigate the sensitivity of each method in detecting the volumetric differences of the hippocampus between the different groups including NC, MCI, and AD, we computed the Cohen’s d effect size based on the corrected hippocampal volumes. Cohen’s d effect size is defined as Cohen’s ${\rm{d}}=\frac{{m}_{1}-{m}_{2}}{S{D}_{Pooled}},$ $S{D}_{Pooled}=\sqrt{\frac{S{D}_{1}^{2}+S{D}_{2}^{2}}{2}},$ where $m$ and $SD$ are the mean and standard deviation respectively ^39^. Based on a conventional operational definition of Cohen’s d, small, medium, and large effect sizes were defined as ${\rm{d}} < 0.5,\,0.5 < {\rm{d}} < 0.8,$ and ${\rm{d}} > 0.8$, respectively^19,39^.

We also carried out classification experiments to distinguish AD patients (n = 36) from NC subjects (n = 29) as well as to distinguish stable MCI (sMCI, n = 11) subjects from progressive MCI (pMCI, n = 21) subjects. The latter was served as an experiment for prediction of MCI conversion. To assess each segmentation method with respect to its classification performance, we trained and tested linear support vector machine (SVM) classifiers^40^ built upon the age of each subject, as well as the left and right hippocampal volume measures derived from its segmentation results. The SVM classifier was built using MATLAB (R2012a) functions with default parameter (C = 1) based on a leave-one-out (LOO) cross-validation procedure. The classification performance was evaluated based on receiver operating characteristic (ROC) curves, summarized by area under the ROC curve (AUC).

## Experimental results

### Parameter optimization results

**Figure 5:**
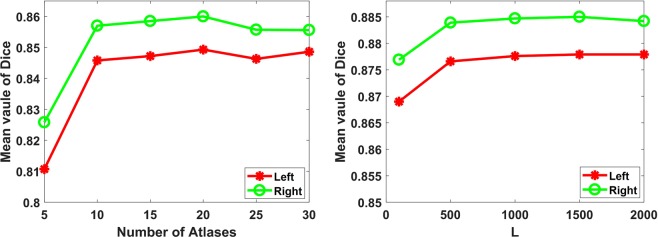
Mean Dice values of segmentation results obtained by majority voting label fusion method with different number of atlases (left), random local binary pattern method with different values of parameter L (right).

The left subfigure of Fig. 5 shows the mean Dice values of segmentation results obtained by majority voting label fusion method with different number of atlases, which illustrates that n = 20 could obtain the optimal segmentation results. The right subfigure of Fig. 5 shows the mean Dice values of segmentation results obtained by the RLBP method with different values L, which demonstrates that the proposed method could perform well when L > 500, and it  could obtain the best segmentation results when L was between 1000 and 1500. Thus, we chose L = 1000 for the computational efficiency. Table 3 shows the average segmentation accuracy of the RLBP method with different parameters measured in terms of the Dice index, and it can be observed that the optimal values were $C={4}^{-4}$ and ${r}_{p}=4$. For the NLP method, the optimal value of ${r}_{p}$ was $1$. For the LLL method, the best parameters were ${r}_{p}=3$ and $\,k=300$. For the JLF method, the best parameters were ${r}_{p}=1$ and $\beta =1$. For the NLW-ML method, the optimal parameters were ${r}_{p}=1$ and $k=9$.

**Table 3:** Dice values (mean ± std) of bilateral hippocampus segmentation results obtained using the RLBP method with different parameters $C$ and ${r}_{p}$

C	${4}^{-5}$	${4}^{-4}$	${4}^{-3}$	${4}^{-2}$	${4}^{-1}$	${4}^{0}$
$${r}_{p}$$						
1	0.8746 ± 0.0432	0.8740 ± 0.0439	0.8668 ± 0.0447	0.8504 ± 0.0446	0.8252 ± 0.0446	0.8040 ± 0.0447
2	0.8798 ± 0.0416	0.8797 ± 0.0416	0.8735 ± 0.0410	0.8571 ± 0.0407	0.834 ± 0.0406	0.8165 ± 0.0406
3	0.8806 ± 0.0408	0.8809 ± 0.0406	0.8748 ± 0.0400	0.8583 ± 0.0392	0.8363 ± 0.0401	0.8179 ± 0.0388
4	0.8807 ± 0.0400	**0**.8813^*^ ± 0.0400	0.8744 ± 0.0394	0.8586 ± 0.0392	0.8358 ± 0.0380	0.8162 ± 0.0373
5	0.8804 ± 0.0395	0.8811 ± 0.0394	0.8750 ± 0.0393	0.8578 ± 0.0383	0.8350 ± 0.0373	0.8150 ± 0.0379

### Segmentation accuracy results

**Figure 6:**
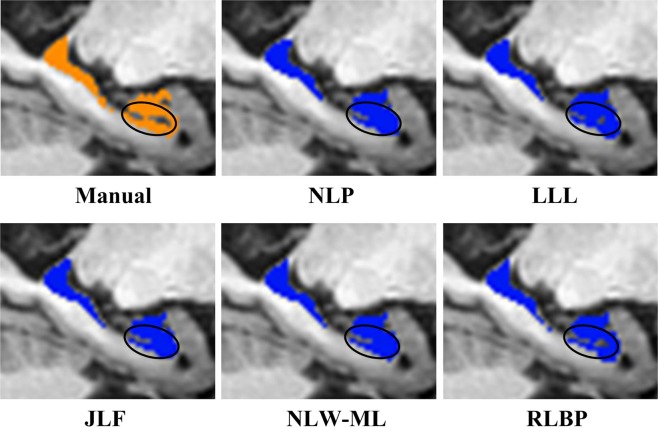
Hippocampus segmentation results of a randomly selected subject using different methods.

**Figure 7:**
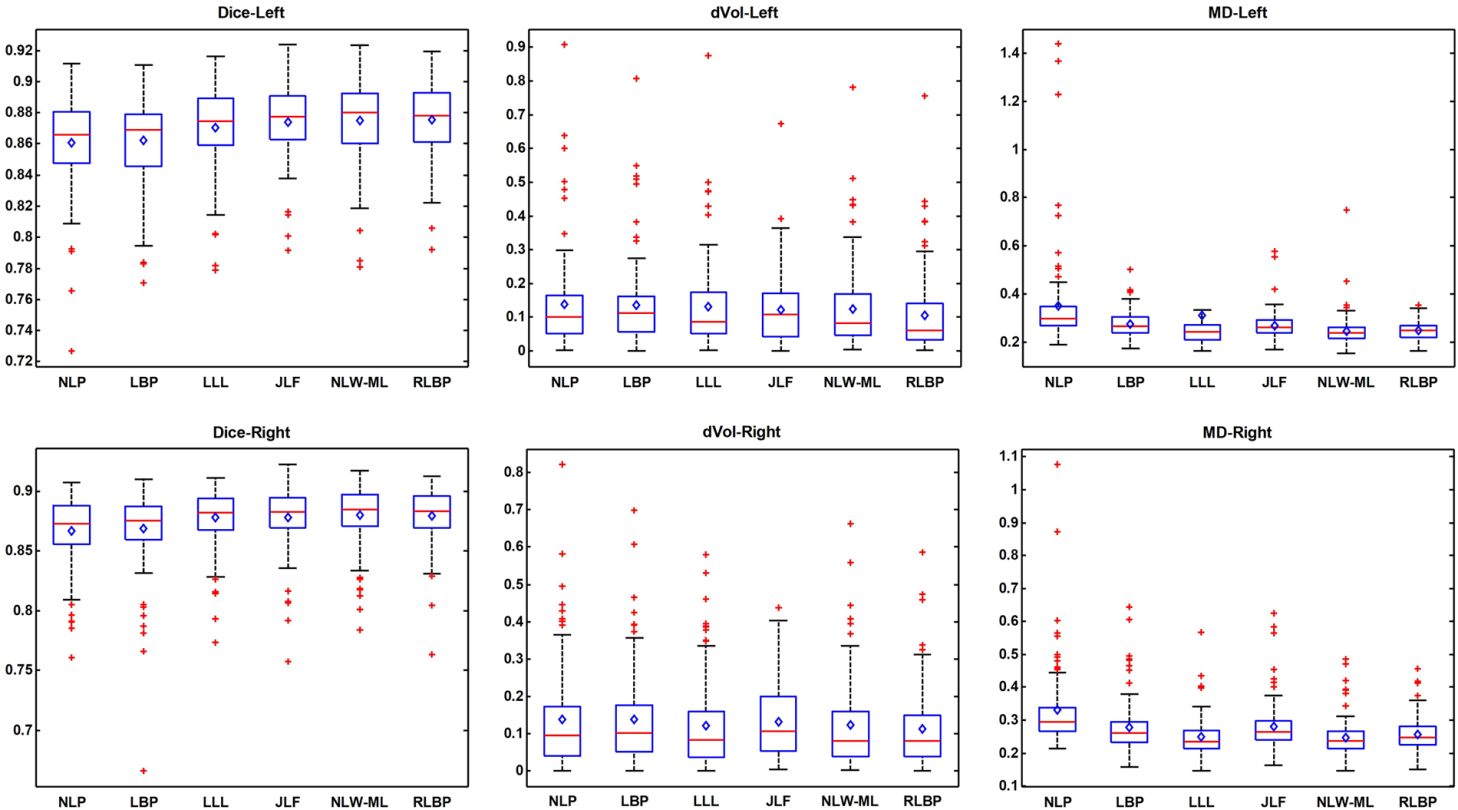
Comparison of various methods for segmenting left hippocampus (top row) and right hippocampus (bottom row) in terms of the Dice, dVol and MD indexes. In each box, the central line is the median, and the central diamond is the mean. The edges of each box are the 25th and 75th percentiles.

**Figure 8:**
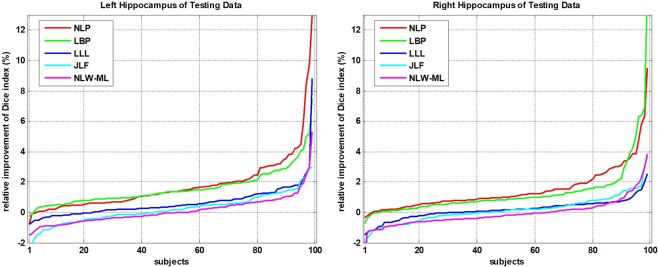
Relative improvement (%) achieved by our method compared with alternative state-of-the-art methods in terms of Dice index values of individual testing images. The relative improvement rates of individual testing images were ranked separately for different methods.

Figure 6 shows a 2D visualization of the segmentation results of a randomly selected subject obtained using different methods, including the NLP, LLL, JLF, NLW-ML and RLBP methods. Table 4 summarizes six segmentation accuracy metrics (mean ± std) of the segmentation results of the test images obtained using the segmentation methods under comparison, including NLP, LBP, LLL, JLF, NLW-ML and RLBP methods. For each metric, the best value was highlighted in bold. The results illustrated that the proposed RLBP method performed statistically better than the NLP, LBP, and LLL methods (pair-wise Wilcoxon signed rank tests, p < 0.05) in most of the metrics used for evaluating their segmentation results. The performance of the RLBP method was comparable to that of the state-of-the-art JLF and NLW-ML algorithms. Figure 7 shows box plots of Dice, dVol and MD indexes of the segmentation results obtained using different methods, while Fig. 8 shows relative improvement of Dice values (%) achieved by RLBP method compared with other label fusion methods. Both of these figures illustrate that the RLBP method achieved competitive performance.

**Table 4:** Six metric index values (mean ± std, p-value) for the segmentation results obtained by different methods (‘*’ indicates RLBP method achieved statistically better results).

		NLP	LBP	LLL	JLF	NLW-ML	RLBP
Dice	left	0.861 ± 0.031* *1*.*0e-10*	0.862 ± 0.027* *1*.*0e-10*	0.870 ± 0.026* *1*.*0e-7*	0.874 ± 0.024 *0*.*063*	0.875 ± 0.025 *0*.*989*	**0**.**876 **±** 0**.**023**
	right	0.867 ± 0.029* *1*.*0e-10*	0.869 ± 0.034* *1*.*0e-10*	0.878 ± 0.024* *0*.*002*	0.878 ± 0.026 *0*.*143*	**0**.**880 **±** 0**.**025** *0*.*014*	0.879 ± 0.024
Jaccard	left	0.757 ± 0.046* *1*.*0e-10*	0.758 ± 0.041* *1*.*0e-10*	0.772 ± 0.040* *1*.*0e-7*	0.777 ± 0.037 *0*.*067*	0.779 ± 0.039 *0*.*986*	**0**.**780 **±** 0**.**035**
	right	0.766 ± 0.043* *1*.*0e-10*	0.769 ± 0.049* *1*.*0e-10*	0.783 ± 0.038* *0*.*002*	0.784 ± 0.040 *0*.*143*	**0**.**787 **±** 0**.**039** *0*.*012*	0.785 ± 0.037
Precision	left	0.870 ± 0.035* *1*.*0e-10*	0.875 ± 0.038* *1*.*0e-7*	0.882 ± 0.036 *0*.*823*	0.872 ± 0.034* *1*.*0e-7*	**0**.**884 **±** 0**.**033** *0*.*146*	**0**.**884 **±** 0**.**030**
	right	0.869 ± 0.041* *1*.*0e-10*	0.871 ± 0.052* *0*.*0004*	0.879 ± 0.039 *0*.*237*	0.867 ± 0.041* *1*.*0e-7*	**0**.**881 **±** 0**.**040** *0*.*007*	0.879 ± 0.036
Recall	left	0.854 ± 0.045* *1*.*0e-10*	0.851 ± 0.036* *1*.*0e-10*	0.860 ± 0.038* *1*.*0e-7*	**0**.**878 **±** 0**.**035** *0*.*0005*	0.867 ± 0.039 *0*.*313*	0.869 ± 0.034
	right	0.867 ± 0.041* *1*.*0e-10*	0.869 ± 0.036* *1*.*0e-10*	0.878 ± 0.033* *0*.*001*	**0**.**891 **±** 0**.**029** *4*.*0e-6*	0.881 ± 0.036 *0*.*709*	0.881 ± 0.033
dVol (cm^3^)	left	0.139 ± 0.145* *1*.*0e-5*	0.136 ± 0.131* *4*.*0e-7*	0.131 ± 0.131* *4*.*0e-6*	0.123 ± 0.107* *0*.*030*	0.124 ± 0.125* *0*.*0009*	**0**.**107 **±** 0**.**118**
	right	0.138 ± 0.140* *0*.*0006*	0.138 ± 0.131* *0*.*001*	0.122 ± 0.121 *0*.*102*	0.132 ± 0.103 *0*.*056*	0.123 ± 0.123 *0*.*086*	**0**.**113 **±** 0**.**111**
MD	left	0.352 ± 0.200* *1*.*0e-10*	0.277 ± 0.055* *1*.*0e-10*	0.313 ± 0.707* *0*.*025*	0.269 ± 0.060* *4*.*0e-6*	**0**.**248 **±** 0**.**067** *0*.*004*	**0**.**248 **±** 0**.**041**
	right	0.332 ± 0.126* *1*.*0e-10*	0.279 ± 0.082* *1*.*0e-7*	0.249 ± 0.059* *2*.*0e-7*	0.280 ± 0.076* *1*.*0e-7*	**0**.**248 **±** 0**.**058** *6*.*0e-7*	0.257 ± 0.051

**Figure 9:**
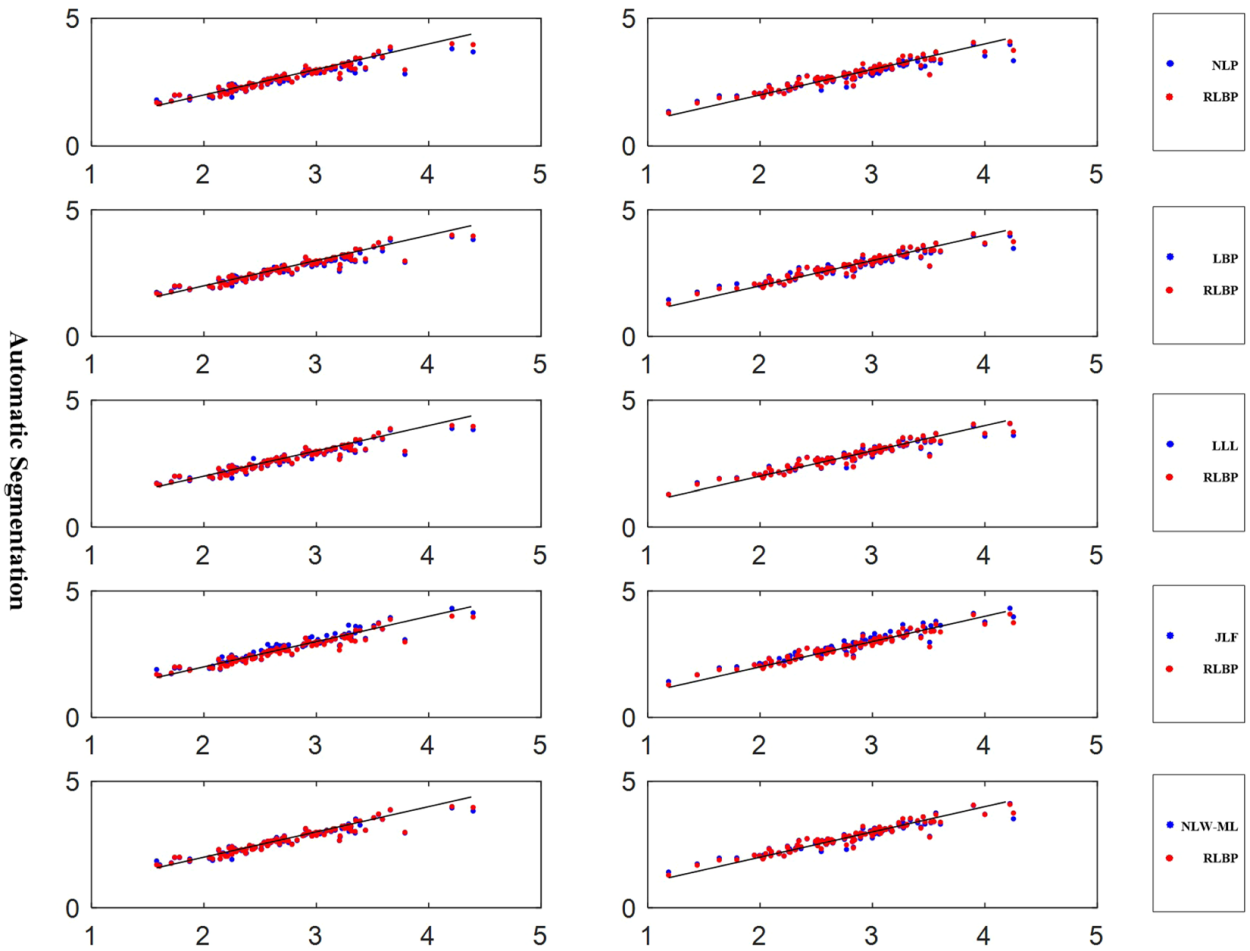
Volumetric comparison of hippocampus segmentation results obtained on using the automatic methods and manual labeling. The black line represents the unity line. From top to bottom: NLP versus RLBP method, LBP versus RLBP method, LLL versus RLBP method, JLF versus RLBP method, and NLW-ML versus RLBP method. The hippocampal volumes were corrected using the total intracranial volumes. The x and y axes are volume measures (cm^3^) of hippocampi segmented by different methods.

Figure 9 shows the scatter plots of the hippocampal volumes estimated using manual segmentation and the automatic segmentation methods, and the correlation coefficients are summarized in Table 5. All automatic segmentation methods obtained the Pearson Correlation coefficients larger than 0.93, with one-tailed p < 0.001.

**Table 5:** Pearson correlation coefficients between hippocampus volumes estimated using manual and automatic methods for both the left and right hippocampi.

	NLP	LBP	LLL	JLF	NLW-ML	RLBP
Left	0.9416	0.9492	0.9467	0.9532	0.9504	**0**.**9595**
Right	0.9395	0.9420	0.9517	**0**.**9610**	0.9493	0.9572

### Hippocampal volumetric analysis results

The corrected volumes of the left and right hippocampi by groups are summarized in Table 6, indicating that the AD subjects had smaller hippocampus than the MCI and NC subjects, and the MCI subjects had smaller hippocampus than the NC subjects. Table 7 summarizes the Cohen’s effect sizes of the hippocampal volumes between various groups. These results indicated that the hippocampal volumes estimated by different methods were sensitive in capturing the differences between AD and NC as well as between MCI and NC groups. However, all the methods, including the manual segmentation method, had median or low effect sizes between the MCI and AD groups. For the left hippocampus, LLL and RLBP methods obtained medium effect sizes (between 0.5 and 0.8), while NLP, LBP, JLF, NLW-ML methods obtained small effect sizes (smaller than 0.5). For the right hippocampus, all methods obtained small effect sizes. However, the proposed RLBP method got the largest effect size.

**Table 6:** Corrected hippocampal volumes (mean ± std) by group (cm^3^).

		Manual	NLP	LBP	LLL	JLF	NLW-ML	RLBP
NC	Left	3.111 ± 0.337	2.971 ± 0.309	2.967 ± 0.291	2.984 ± 0.308	3.111 ± 0.319	3.015 ± 0.307	3.029 ± 0.310
	Right	3.151 ± 0.324	3.076 ± 0.294	3.101 ± 0.296	3.1145 ± 0.300	3.224 ± 0.314	3.130 ± 0.300	3.143 ± 0.316
MCI	Left	2.657 ± 0.484	2.567 ± 0.457	2.581 ± 0.471	2.602 ± 0.465	2.668 ± 0.528	2.594 ± 0.473	2.615 ± 0.483
	Right	2.716 ± 0.543	2.678 ± 0.472	2. 700 ± 0.481	2.704 ± 0.493	2.774 ± 0.549	2.702 ± 0.497	2.727 ± 0.513
AD	Left	2.403 ± 0.532	2.353 ± 0.415	2.361 ± 0.434	2.352 ± 0.434	2.436 ± 0.489	2.367 ± 0.436	2.365 ± 0.454
	Right	2.528 ± 0.576	2.500 ± 0.435	2.531 ± 0.446	2.524 ± 0.477	2.602 ± 0.502	2.521 ± 0.456	2.516 ± 0.485

**Table 7:** Cohen’s d effect sizes between the three diagnosis groups (NC-MCI, NC-AD, and MCI-AD).

		Manual	NLP	LBP	LLL	JLF	NLW-ML	RLBP
NC-MCI	Left	**1**.**0889**	1.0348	0.9855	0.9682	1.0144	1.0566	1.0205
	Right	0.9730	1.0131	1.0062	1.0061	1.0071	**1**.**0434**	0.9781
NC-AD	Left	1.5913	1.6888	1.6388	1.6808	1.6344	**1**.**7172**	1.7092
	Right	1.3329	1.5542	1.5052	1.4823	1.4848	**1**.**5783**	1.5309
MCI-AD	Left	0.4996	0.4919	0.4849	**0**.**5563**	0.4562	0.4977	0.5333
	Right	0.3355	0.3928	0.3633	0.3721	0.3252	0.3799	**0**.**4209**

**Figure 10:**
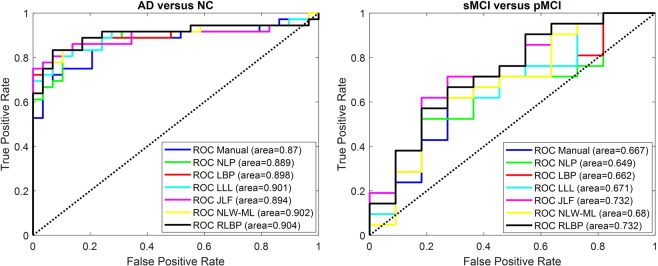
ROC curves of the SVM classifiers built upon the age of each subject and hippocampal volume measures estimated by different methods under comparison. Left panel shows ROC curves for distinguishing AD patients from NC subjects, right panel shows ROC curves for distinguishing sMCI from pMCI subjects.

The performance of different SVM classifiers built upon the age of each subject, as well as the left and right hippocampal volume measures estimated by different methods is shown in Fig. 10. DeLong statistical test was further used to compare the ROC curve of the RLBP method with other methods^41^. Table 8 lists the AUC values, their standard errors, and p-values of the statistical tests. It can be observed that the RLBP method obtained the best AUC value for distinguishing AD from NC subjects, and the JLF and RLBP methods obtained the best AUC for distinguishing pMCI from sMCI subjects. In particular, the RLBP method was better than NLP, LBP and NLW-ML in distinguishing pMCI from sMCI subjects (p < 0.05).

**Table 8:** AUC values ± standard errors, p-values of the SVM classifiers built upon the age of each subject and hippocampal volume measures estimated by different methods under comparison (‘*’ indicates RLBP method achieved statistically better results).

	Manual	NLP	LBP	LLL	JLF	NLW-ML	RLBP
NC-AD	0.870 ± 0.047 *0*.*151*	0.889 ± 0.044 *0*.*064*	0.898 ± 0.043 *0*.*528*	0.901 ± 0.041 *0*.*661*	0.894 ± 0.045 *0*.*437*	0.902 ± 0.042 *0*.*529*	**0**.**904 **±** 0**.**042**
sMCI- pMCI	0.667 ± 0.104 *0*.*307*	0.649 ± 0.103* *0*.*033*	0.662 ± 0.105* *0*.*037*	0.671 ± 0.105 *0*.*126*	**0**.**732 **±** 0**.**099** *1*.*000*	0.680 ± 0.107* *0*.*019*	**0**.**732 **±** 0**.**098**

### Computational cost

The NLP, LLL, NLW-ML, and RLBP methods were implemented using MATLAB 7.14 on a personal computer with a four-core 3.4-GHZ CPU, and the JLF method was implemented using C +  + . On average, the RLBP method required approximately 8.0 min to segment one side of the hippocampus, while the NLP, LLL, NLW-ML, and JFL methods required approximately 4.0 min, 6.0 min, 20 min, and 0.5 min respectively to segment one side of the hippocampus. Note that, this is the time for feature extraction and segmentation excluding image registration.

## Discussion and conclusions

Machine learning based multi-atlas label fusion methods have obtained great success in a variety of image segmentation problems. In these methods, feature extraction plays an important role^10,27^. In this study, we presented an RLBP feature extraction method for machine learning based label fusion. In contrast to the original LBP feature extraction method, the proposed RLBP method computes a large number of random combinations before the binarization. It is known that a small pixel difference is vulnerable to image noise, which may degrade the pattern recognition performance when the LBP features are used to build prediction models as the LBP method treats large and small differences in the same way. Benefiting from the random combination processing, the large difference will contribute more in the binarized features, which makes the proposed RLBP method more robust to image noise than LBP method. The random combination weights in the RLBP feature extraction method can be seen as random texture feature filters. Using a large number of random texture feature filters, the statistical properties of the image patches can be captured efficiently. The experiment results demonstrated that the proposed RLBP feature extraction method was more effective than the LBP method when using the same linear regression model in multi-atlas based hippocampus segmentation.

The comparison experiments with start-of-the-art multi-atlas label fusion methods demonstrated that the proposed RLBP method exhibited superior or comparable segmentation results, which were evaluated using by a variety of image segmentation metrics. It is worth noting that hippocampal volumes estimated by the automatic segmentation methods were highly correlated with the manual labeling results. The volumetric analysis experiments demonstrated that all the hippocampus segmentation methods under comparison achieved promising performance for distinghusing NC from AD and MCI subjects based on their hippocampal volume measures. We further used a linear SVM classifier with the age of each subject as well as left and right hippocampal volume measures estimated by each method as features to distinguish AD patients from NC subjects (diagnosis study) and distinguishing sMCI from pMCI subjects (prognosis study). The results showed that the RLBP method obtained the best AUC values for distinguishing AD from NC subjects, as well as for distinguishing pMCI from sMCI subjects. However, because of the limited samples, the RLBP method was only statistically better than NLP, LBP and NLW-ML for distinguishing pMCI from sMCI subjects (p < 0.05).

In the present study, linear regression models were built by using RLBP features to achieve multi-atlas label fusion. In our experiments, we also tested sparse linear SVM classifiers with RLBP features for multi-atlas label fusion. However, the results were not as good as thosed obtained by the linear regression models. Compared with training a nonlinear SVM classification model^27,33^, the computational cost of training a linear regression model is much lower. Thus, the proposed RLBP method was faster than the existing nonlinear SVM classification based label fusion methods^27,33^. The RBLP based label fusion method achieved a segmentation accuracy similar to NLW-ML with a faster computation speed^33^. The proposed method could be further improved by incorporating deep learning techniques in order to extract more discriminative image features^42–54^.

The proposed method is a learning-based technique, and therefore, its performance is bounded by the quality of the training and testing data. In this study, we adopted the EADC-ADNI dataset for both training and testing^35^. Particularly, 68 1.5 T and 67 3 T volumetric structural ADNI scans from different subjects were segmented using five qualified harmonized protocol tracers, the absolute interrater intraclass correlation coefficients of which were 0.953 and 0.975 (left and right).

As a multi-atlas segmentation method, a major issue is the high computational cost of nonlinear image registration. To reduce the computational cost, several methods have been proposed, such as the enhanced atlas-based segmentation method^55^, optimized patch match label fusion method^56^, and multi-atlas learner fusion method^57^. In this study, we adopted an atlas selection strategy for selecting the most similar atlases for reducing the computational cost of the nonlinear image registration^9,10^. However, it would be interesting to combine the proposed method with the enhanced atlas based segmentation method, the optimized patch match label fusion method, and the multi-atlas learner fusion method to further improve the computational speed. A very promising direction for improving both the computational efficiency and segmentation accuracy by utilizing deep learning techniques has been reported in recent papers^42–54^. By using deep learning techniqures, more discriminative image features can be extracted to achieve improved segmentation performance.

In conclusion, we have proposed a novel RLBP method to extract image features for building prediction models to fuse labels in the framework of multi-atlas segmentation. The results of the evaluation experiments showed that the proposed RLBP method could achieve hippocampus segmentation accuracy competitive to or comparable with that of state-of-the-art label fusion methods.
